# The undervalued self: social class and self-evaluation

**DOI:** 10.3389/fpsyg.2014.01404

**Published:** 2014-12-05

**Authors:** Michael W. Kraus, Jun W. Park

**Affiliations:** ^1^University of Illinois at Urbana-Champaign, Champaign, IL, USA; ^2^Pomona College, Claremont, CA, USA

**Keywords:** social class, self-concept, emotion, self-esteem, socioeconomic status

## Abstract

Social class ranks people on the social ladder of society, and in this research we examine how perceptions of economic standing shape the way that individuals evaluate the self. Given that reminders of one’s own subordinate status in society are an indicator of how society values the self in comparison to others, we predicted that chronic lower perceptions of economic standing vis-à-vis others would explain associations between objective social class and negative self-evaluation, whereas situation-specific reminders of low economic standing would elicit negative self-evaluations, particularly in those from lower-class backgrounds. In Study 1, perceptions of social class rank accounted for the positive relationship between objective material resource measures of social class and self-esteem. In Study 2, lower-class individuals who received a low (versus equal) share of economic resources in an economic game scenario reported more negative self-conscious emotions—a correlate of negative self-evaluation—relative to upper-class individuals. Discussion focused on the implications of this research for understanding class-based cultural models of the self, and for how social class shapes self-evaluations chronically.

Since the late 1970s, the United States has fostered tremendous growth for the top earners in the country: the highest one per cent saw an increase in their pretax income share from around 9% in 1978 to nearly 23% by 2012, and by current estimates, the top decile nets close to 50% of all market income in the US (Saez, 2013). A growing body of research in the social sciences reveals that levels of economic inequality are associated with societal well-being: relative to more equal countries of similar economic development, such as Denmark and Norway, highly unequal countries like the United States suffer from higher rates of obesity, imprisonment, and mental illness among many other important health and social concerns (Wilkinson and Pickett, 2006). Moreover, these negative effects on well-being are heightened particularly for those at the lowest levels of the social class hierarchy—with fewer material and social resources (Wilkinson, 1996; Wilkinson and Pickett, 2009). When economic inequality deepens, those at the bottom of society’s hierarchy suffer the most.

In this research, we examine how *perceptions* of one’s own economic standing—chronically reported or specific to a situation—explain how social class is related to patterns of self-evaluation. We make the following predictions: *chronic* perceptions of social class rank relative to others will explain associations between assessments of actual economic resources and negative self-evaluation, whereas *situation-specific* reminders suggesting one’s lower economic position relative to others will elicit processes related to negative self-evaluation (i.e., enhanced negative self-conscious affect), particularly for individuals with fewer actual economic resources.

## PERCEPTIONS OF ECONOMIC STANDING

Social class (socioeconomic status or SES) is typically defined as the experience of contrasting levels of objective economic and social resources, and measured using indices of educational attainment, annual income, and occupation status (Oakes and Rossi, 2003; Kraus et al., 2012; Kraus and Stephens, 2012; Stephens et al., 2012b). Together, education, income, and occupation status represent the material substance of social class and shape the life-trajectories of individuals in profound ways—even shortening the life course for those at the bottom of the class hierarchy, relative to those above them (Adler et al., 1994).

Recent theoretical advances reveal that the objective material aspects of social class shape how individuals perceive their own economic position in the social class hierarchy vis-à-vis others (Kraus et al., 2009). Specifically, individuals rank themselves within their small social groups, local community, and society at large by comparing their own income, education, and occupation status to that of others (for a review, see Kraus et al., 2013b). This ranking process is facilitated by the capacity for individuals to accurately assess the social class of others during brief social interactions (Kraus and Keltner, 2009), and by the tendency for individuals to share accurate information about the self in order to facilitate social interaction (e.g., Ambady and Rosenthal, 1992).

Aligning with research suggesting the importance of social rank for the social lives of non-human mammals (Sapolsky, 2005), the acute awareness of one’s own economic position vis-à-vis others elicits important changes in health and well-being: in a series of studies, participants estimated their social class rank by placing an “X” on a ten-rung ladder representing ascending levels of income, education, and occupation status in society, and then underwent measures of various health indicators. Across studies, perceptions of lower social class rank predicted elevated blood pressure (Adler et al., 2000; Wright and Steptoe, 2005), greater susceptibility to cold-causing viruses (Cohen et al., 2008), and increased risk for mortality (Kopp et al., 2004) relative to perceptions of upper-class rank. Moreover, in each of these studies, subjective perceptions of position on the social ladder predicted health outcomes independently of objective material resource measures of social class (i.e., annual income, educational attainment).

Importantly, people come to understand their economic standing in society chronically, and also adjust these perceptions to fit the current situation or context (for a review, see Kraus et al., 2012). Evidence for chronic perceptions of economic standing comes from research indicating that social class rank predicts poor self-rated health and negative affect independently of one’s current mood state (Kraus et al., 2013a). Evidence for the situation-specific nature of perceptions of economic standing comes from research indicating that people will shift their perceptions of social class position as a result of anticipating an interaction with someone above or below them in the class hierarchy (e.g., Kraus et al., 2010). For instance, one study found that thinking of people at the top of the social class hierarchy led individuals to report themselves as having a lower class position in society, and to report more hostile affect in response to an ambiguously threatening social situation, relative to thinking of someone at the bottom of the hierarchy—presumably because perceptions of lower social class rank in the former case elicit heightened threat reactivity (Kraus et al., 2011).

Overall, the above research suggests that perceiving oneself as lower in economic standing relative to others has profound implications for health and well-being across the life course. In the present research, we consider how chronic and situation-specific perceptions of economic standing, that arise from the material conditions of the lives of individuals, shape positive or negative evaluations of the self-concept.

## THE UNDERVALUED SELF

The way that individuals evaluate the self as positive or negative is subject to a variety of social factors including cultural background (Markus and Kitayama, 1991), the presence of significant others (Baldwin, 1992; Bosson and Swann, 1999), the threat of others’ evaluations (James, 1890; Sedikides and Gregg, 2008), and the salient goals elicited by the social context (Shah, 2003). Though much research suggests that social class profoundly shapes aspects of the social self (c.f., Snibbe and Markus, 2005; Stephens et al., 2007, 2012b; Kraus et al., 2012), how social class influences self-evaluation has not, in our estimation, been well understood in psychology research.

We contend that perceptions of economic standing will influence self-evaluation in two ways: chronic perceptions of one’s lower economic standing relative to others, we predict, will explain associations between negative self-evaluation and lower objective material resource measures of social class (Hypothesis I). In contrast, situation-specific information suggesting that one is lower in economic resources relative to others will elicit lower self-evaluation, particularly for those from relatively lower social class backgrounds (Hypothesis II).

With respect to chronic perceptions of economic standing, individuals from lower class backgrounds consistently develop lower perceptions of their economic resources relative to others (Adler et al., 2000; Kraus et al., 2012), and it is these perceptions, we predict, that drive self-evaluation: specifically, those who perceive themselves as being at the bottom of society’s economic hierarchy are acknowledging the lower value of the self, in economic terms, relative to other individuals. In contrast, those perceiving the self as being at the top of society’s economic hierarchy are reporting the self’s enhanced value. We reason that judgments of the economic value of the self will carry over into other self-relevant domains, and will shape more general self-evaluations.

Studies supporting the relationship between social class and self-evaluation are limited but suggestive: for instance, in a meta-analysis of 446 samples, a small but consistent positive association between self-esteem and objective resource measures of social class was observed—suggesting that lower-class individuals have lower self-esteem than their upper-class counterparts (Twenge and Campbell, 2002). As well, objective material resource measures of social class correlate negatively with measures of dysphoric affect in both university students (Kraus et al., 2011) and in adults (Adler et al., 2000), indicating that lower-class individuals feel more down and depressed relative to their upper-class counterparts. As well, first generation college students—whose parents never attended a 4 year university—experience more negative self-conscious emotions (i.e., guilt) about leaving their families to attend university than do students whose parents attended 4 year universities in the past (Covarrubias and Fryberg, 2014). As the experience of negative self-conscious emotion is a correlate of self-evaluation, these findings are suggestive evidence in support of our predictions.

Our contention that situation-specific perceptions of low economic resources will predict more negative self-evaluations, particularly for individuals from lower-class backgrounds arises from the following reasoning: contexts should elicit increased self-evaluation in lower-class individuals because they remind these individuals of their chronic lower share of economic resources in society. In contrast, for upper-class individuals, contexts that provide low economic resources have no bearing on chronic economic states and as a result, will not elicit negative evaluations of the self.

Research examining other low status groups in society is suggestive of this prediction: being a member of a low status group tends to be threatening for individuals, particularly when they are made aware of their own subordinate status by situational cues: in the literature on stereotype threat, low status groups in society (e.g., African Americans) tend to perform more poorly on tests of math ability only to the extent that they have been made aware of their low status identity prior to the test-taking (e.g., Steele and Aronson, 1995). In an example related to social class, research on university students found that middle class students performed more poorly on executive functioning tasks only when they were reminded of their lower level of economic resources when compared to other members of the academic community (e.g., faculty, upper-class students; Johnson et al., 2011).

## THE PRESENT RESEARCH

In the present research we report two studies that examine our hypotheses related to social class rank perceptions and self-evaluation. Specifically, we predict that chronic perceptions of social class rank will explain associations between objective social class and self-evaluation (Hypothesis I), whereas situation-specific perceptions of low economic resources will elicit negative self-evaluations particularly for individuals from lower-class backgrounds (Hypothesis II). We test these predictions across two studies using correlational (Study 1) and experimental (Study 2) assessments of perceptions of economic resources. In particular, Study 1 examines associations between social class background, subjective social class, and self-esteem—a global assessment of self-evaluation—while controlling for trait neuroticism, a predictor of self-esteem in prior research (e.g., Judge et al., 2002). We controlled for trait neuroticism in order to determine the unique influence of social class rank on self-evaluation that is separate from trait-related patterns of emotion responding. We expected chronic perceptions of social class rank to mediate associations between objective social class and self-esteem. In Study 2, university undergraduates were exposed to an economic game with a partner wherein they were manipulated to receive either an equal or low share of economic resources prior to evaluating the self. In Study 2, we assessed negative self-conscious affect because of its correlation, in prior research, to situation-specific self-evaluation. Emotions are brief affective experiences with specific eliciting events (Keltner and Lerner, 2010), and self-conscious emotions are brief assessments of positive or negative feelings about the self (Tangney et al., 2007b). We expected individuals from lower-class backgrounds to report more negative self-conscious emotions particularly following their exposure to a low (versus equal) share of economic resources.

## STUDY 1: CHRONIC PERCEPTIONS OF ECONOMIC STANDING AND SELF-ESTEEM

In Study 1 we asked a sample of online participants to provide information about their own objective material resources, perceptions of their economic standing—measured in terms of subjective social class rank, and their self-esteem. We expected that the association between material resource measures of social class and self-esteem would be statistically accounted for, in part, by perceptions of social class rank. As well, we expected this effect to persist even after accounting for a potential alternative explanation of associations between social class and self-esteem. Self-esteem is typically highly correlated with trait neuroticism (Judge et al., 2002) and so we sought to determine if the association between social class rank and self-esteem was independent of this personality factor.

### MATERIALS AND METHODS

#### Participants

Participants were 744 adults recruited online through Amazon Mechanical Turk. On average, participants were 32 years of age (SD = 11.94), and the majority of the sample was male (*n* = 378). Participants were all residents of the United States and self-identified as European American (*n* = 563), African American (*n* = 56), Asian American (*n* = 65), Latino (*n* = 36), or listed other as their ethnic background (*n* = 37). Participants were permitted to select more than one ethnic category. The research was approved by the review board for research on human subjects at the University of Illinois.

#### Procedure

Participants accessed a survey about their important social groups and first filled out demographic information about their social class as part of a larger research project. Finally, participants filled out personality measures and a measure of self-esteem, were probed for suspicion, debriefed about the hypothesis of the study, and compensated $1 for their participation.

#### Measures

***Social class.*** We assessed participant education and income as objective material resource measures of social class (Kraus and Stephens, 2012). Income was measured by averaging participant self-reports of current annual income and annual family income, each assessed using eight categories: (1) <*$15,000*, (2) *$15,001–$30,000*, (3) *$30,001–$45,000*, (4) *$45,001–$60,000*, (5) *$60,001–$75,000*, (6) *$75,001–$100,000*, (7) *$100,001–$150,000*, and (8) >*$150,000*. The median income of the sample was between $30,001 and $45,000 which is consistent with US national median levels (www.census.gov; *M* = 3.32, SD = 1.58). Participant education was assessed using four categories: (1) *less than high school graduation*, (2) *high school graduation*, (3) *college graduation*, and (4) *post-graduate degree* (*M* = 2.64, SD = 0.70). To create an overall composite of social class, we standardized and averaged the income composite and education variables to create an overall measure of objective social class (*M* = –0.001, SD = 0.83).

As in prior research (Kraus et al., 2009), we assessed perceptions of social class rank using Adler et al. (2000) measure of subjective SES. In this measure, participants indicated their position on a 10-rung ladder representing ascending levels of income, education, and occupation status in society (*M* = 5.24, SD = 1.80).

***Self-esteem.*** The 10-item Rosenberg self-esteem scale was used for our measure of self-esteem (Rosenberg, 1965). Items were assessed on a 7-point Likert scale (–3 = *strongly disagree*, 3 = *strongly agree*; *M* = 1.21, SD = 1.29, α = 0.94). A sample item is “I feel that I have a number of good qualities.”

***Neuroticism.*** Participants filled out the 44-item Big Five Inventory using 5-point Likert scales (1 = *Strongly disagree*, 5 = *Strongly agree*; John and Srivastava, 1999). Neuroticism was assessed using 8 items (e.g., I see myself as anxious, easily upset; *M* = 2.76, SD = 0.87, α = 0.88).

### RESULTS

Correlations between key variables are displayed in Table 1. As expected, all measures of social class were moderately inter-correlated—with subjective social class rank moderately positively associated with annual income and educational attainment. Aligning with our first hypothesis, income and the composite social class measure showed small positive associations with self-esteem. Notably, the direction and magnitude of the relationship between these indices of social class and self-esteem is consistent with prior meta-analytic estimates (Twenge and Campbell, 2002). Trait neuroticism was significantly negatively associated with social class rank, the objective composite of social class, income, and self-esteem. Aligning with our expectations, elevated social class rank was significantly positively associated with elevated self-esteem, suggesting that perceptions that one has elevated social class rank in society are associated with more general positive evaluations of the self.

**Table 1:** Correlations between social class, self-esteem, and trait neuroticism (Study 1).

	Self-esteem	Social class rank	Income	Education	Class composite
Self-esteem	–				
Social class rank	0.30*	–			
Income	0.14*	0.34*	–		
Education	0.05	0.25*	0.36*	–	
Class composite	0.12	0.35*	0.83*	0.83*	–
Neuroticism	−0.67*	−0.21*	−0.06	−0.05	−0.11*

*p < 0.05.

The zero-order correlation analysis shows predicted associations between social class and self-esteem, but it cannot test Hypothesis I: perceptions of social class rank vis-à-vis others will statistically account for the relationship between objective material resource measures of social class and self-esteem. To directly test this hypothesis, we conducted a mediation path analysis with the composite measure of objective material resources of social class as the predictor variable, self-esteem as the outcome variable, and perceptions of social class rank as the mediator (Baron and Kenny, 1986).

**FIGURE 1:**
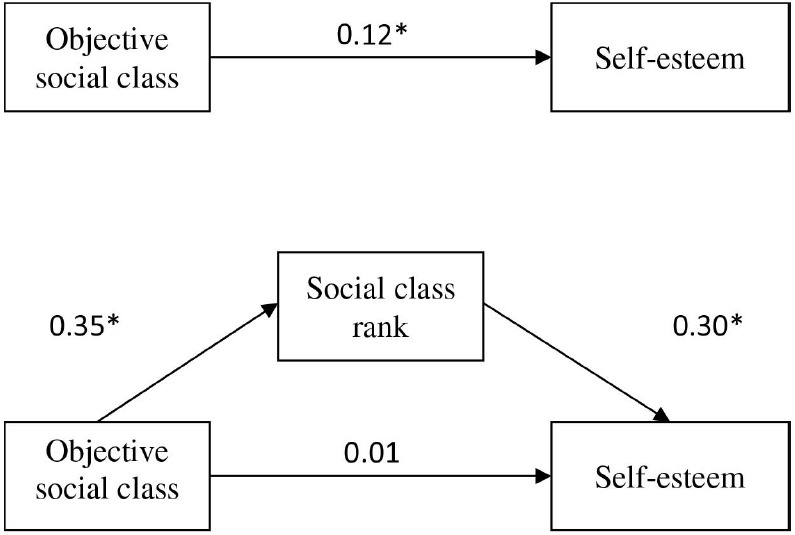
**Path analysis showing the relationships between objective social class, perceptions of social class rank, and self-esteem (Study 1).** The top panel shows the association between objective social class, measured in terms of a composite of educational attainment and annual income, and self-esteem. The bottom panel shows the association between objective social class and self-esteem, through subjective social class rank. Numbers indicate standardized beta coefficients. *p < 0.05.

The results of this path analysis are both described below and displayed in Figure 1. Consistent with our first hypothesis, the composite measure of objective social class was positively related to self-esteem, with relatively upper-class individuals reporting higher self-esteem than their comparatively lower-class counterparts, β = 0.12, *t*(738) = 3.20, *p* < 0.01. Moreover, when accounting for the significant relationships between subjective social class rank, objective social class, β = 0.35, *t*(739) = 10.25, *p* < 0.01, and self-esteem, β = 0.30, *t*(737) = 8.02, *p* < 0.01, the originally significant relationship between objective social class and self-esteem was reduced, *β* = 0.01, *t*(737) = 0.29, *p* = 0.77. A bootstrapping procedure using 2,000 re-samples revealed a positive indirect effect of objective social class on self-esteem through social class rank *b* = 0.17, SE = 0.03, 95% CI (0.12–0.22; Preacher and Hayes, 2004, 2008). This indirect effect provides correlational evidence in support of Hypothesis I—that the association between objective social class and self-esteem would be explained, in part, by perceptions of social class rank.^1^

We also conducted this same mediation path analysis while accounting for trait neuroticism. This latter path analysis yielded similar findings: the composite measure of objective social class was positively related to self-esteem β = 0.06, *t*(716) = 2.05, *p* < 0.05. Moreover, when accounting for the significant relationships between subjective social class rank, objective social class β = 0.34, *t*(716) = 9.83, *p* < 0.01, and self-esteem β = 0.17, *t*(715) = 5.67, *p* < 0.01, the originally significant relationship between objective social class and self-esteem was reduced, β = 0.00, *t*(715) = 0.01, *p* = 0.99. A bootstrapping procedure using 2000 resamples revealed a positive indirect effect of objective social class on self-esteem through social class rank *b* = 0.09, SE = 0.02, 95% CI (0.05–0.13; Preacher and Hayes, 2004, 2008). Neuroticism was negatively associated with self-esteem in the path analysis β = –0.64, *t*(715) = –22.99, *p* < 0.05.

### DISCUSSION

The results of Study 1 provide correlational evidence in support of our first hypothesis: specifically, people from lower objective social class backgrounds have reduced self-esteem relative to their upper-class counterparts, and this association is statistically explained by subjective perceptions of social class rank in comparison to others. Moreover, these findings were observed after accounting for trait neuroticism which is negatively associated with self-esteem.

As the above evidence is correlational, the direction of the relationships between variables in the current analysis cannot be determined—which raises the alternative possibility that low self-evaluations lead to lower perceptions of position on the social class hierarchy. Study 2 uses an experimental paradigm to test Hypothesis II: reminders of one’s lower position in the social class hierarchy elicit negative self-evaluation particularly among lower-class individuals.

## STUDY 2: SOCIAL CLASS, NEGATIVE SELF-CONSCIOUS AFFECT, AND RESOURCE SHARING

In Study 2, we test the hypothesis that lower-class individuals experience more negative evaluations of the self, relative to upper-class individuals, particularly when reminded of their lower economic standing. Specifically, we expected that lower-class individuals would react with elevated self-conscious emotion to low, rather than equal, resource sharing by a stranger—because such sharing would remind them of their chronic lower positions in the social class hierarchy. In contrast, we expected that upper-class individuals would not show this pattern of emotion reactivity because low resource sharing has no relationship to their chronic perceptions of social class rank. To that end, we used an economic game interaction in which participants, varying in family social class, were manipulated to receive a very low or a nearly equal share of economic resources from an anonymous partner. In addition, given that personality factors like trait neuroticism predict negative self-evaluations (Judge et al., 2002), we once again sought evidence suggesting that associations between social class rank and self-evaluation are independent of personality-related patterns of affective responding.

### MATERIALS AND METHODS

#### Participants

Participants were 103 university students recruited for course credit at a major public University in the western United States. Participants self-identified as European American (*n* = 17), African American (*n* = 5), Asian American (*n* = 51), Latino (*n* = 8), Native American (*n* = 6), or listed other as their ethnic background (*n* = 17). The research was approved by the University review board for research on human subjects.

#### Procedure

Participants arrived at the experiment individually and were seated at one of five closed computer cubicles. Participants were instructed that they would be playing an economic game where they would have the opportunity to earn raffle tickets which would be used at the end of the semester to win gift certificates to an online retailer. The experimenter then instructed participants to start filling out their own demographic information, including measures of family social class, baseline emotions, and personality.

Next, participants started an economic game, ostensibly with an experiment partner in one of the four other cubicles in the room. Participants were told that the game was a Dictator game (Camerer and Thaler, 1995) and that they would be playing the part of the recipient. Their partner would be the distributor, and would have the chance to allocate a total of 10 raffle tickets between himself or herself and the participant. In the Dictator Game, the recipient must accept the distributor’s allocation no matter how high or low it is.

Following these instructions, participants waited on a loading screen as the distributor ostensibly decided how to allocate the raffle tickets. After waiting for 30 s, the participants were informed of the distributor’s decision: in the near equal sharing condition, the distributor shared four tickets out the 10 with the participant. In the low sharing condition, the distributor shared one ticket out of the 10 with the participant. After electronically receiving these tickets participants filled out ratings of their own emotions, were given an opportunity to engage in the Dictator game as the distributor with the same partner (to test the salience of our economic sharing manipulation), were probed for suspicion, and were debriefed about the hypotheses of the study. At the end of data collection a raffle was held and three gift certificates of $25 to Amazon.com were distributed to participants.

#### Measures

***Social class.*** As in prior research (Kraus and Keltner, 2009), the social class of participants was assessed using annual family income and educational attainment of participants’ parents. Annual family income was assessed using six categories: (1) <*$15,000*, (2) *$15,001–$45,000*, (3) *$45,001–$60,000*, (4) *$60,001–$75,000*, (5) *$75,001–$100,000*, and (6) >*$100,000*. The median family income of the sample was between $60,001 and $75,000 (*M* = 3.79, SD = 1.69). Educational attainment was assessed using three categories: (1) *high school graduation*, (2) *college graduation*, or (3) *advanced degree completion*. The sample contained participants with both mothers (*n* = 17; *M* = 2.41, SD = 0.74) and fathers (*n* = 16; *M* = 2.35, SD = 0.75) with high school graduation as their highest level of education. To calculate social class, participants’ scores on parental income and education were standardized and averaged to create a composite measure of family social class (*M* = 0.00, *SD* = 1.00).

***Emotion ratings.*** In Study 2, we assessed self-conscious emotions as a correlate of negative self-evaluation and related measures. Participants self-rated 23 emotions at baseline and then again directly following the economic game. The emotions were amusement, anger, awe, compassion, contempt, contentment, desire, disgust, embarrassment, excitement, fear, guilt, happiness, hope, inspiration, interest, jealousy, love, relaxation, sadness, arousal, surprise, and worry. Participants responded on an 8-point Likert scale (0 = *not at all*, 8 = *very much*). We created a composite for self-conscious emotions (embarrassment, fear, guilt, and worry; α_time1_ = 0.71, α_time2_ = 0.84), anger (anger, contempt, and disgust; α_time1_ = 0.76, α_time2_ = 0.76), and overall negative emotion (anger, contempt, disgust, embarrassment, fear, guilt, worry, jealousy, and sadness; α_time1_ = 0.88, α_time2_ = 0.87). We were interested in changes in self-conscious (*M* = –0.29, SD = 0.87), anger (*M* = 0.74, SD = 1.31), and negative (*M* = 0.14, SD = 0.74) emotions following the economic game.

***Neuroticism.*** The ten item personality inventory (Gosling et al., 2003) was used to assess participant neuroticism. Participants responded to two items (e.g., “I see myself as anxious, easily upset.”) using 7-point Likert scales (1 = *disagree strongly*, 7 = *agree strongly*; *M* = 3.18, SD = 1.17).

***Dictator game.*** Participants were given the opportunity to play the Dictator game a second time with the same partner, but in this second round they played the role of distributor and allocated as many as 10 tickets to their partner (*M* = 4.36, SD = 1.51). We used this second Dictator game to assess the efficacy of our manipulation of economic sharing.

### RESULTS

#### Manipulation check

We expected that our manipulation of resource sharing would make participants aware of their lower economic standing within the interaction. To determine if our manipulation was successful in shifting current economic standing within the interaction, we first sought to determine if participants changed their subsequent Dictator game behavior as a distributor, based on their experience as a recipient. We expected that an initial low share of resources would engender reciprocity (i.e., lower resource sharing in return) on the part of participants in the second game (Camerer and Thaler, 1995). Results support this pattern: participants shared more with the partner who had allocated them a near equal (*M* = 4.83) relative to a low (*M* = 3.92) share of the ticket resources *t*(101) = –3.18, *p* < 0.05. Participant social class and gender were both unassociated with allocation decisions in the second Dictator game. This evidence supports the assertion that participants were aware of their own interaction-specific economic standing.

#### Social class, resource sharing, and changes in emotion

We predicted that lower-class individuals would experience increases in negative self-conscious emotions when confronted with low, relative to equal, sharing of economic resources—because such resource sharing practices remind lower-class individuals of their chronic lower position in the social class hierarchy. We tested this hypothesis by conducting a linear regression analysis predicting self-conscious affect at time 2 following the first Dictator game. We entered the sharing condition (coded as “–1” for low and “1” for equal), participant social class, self-conscious emotion at baseline, and the interaction between participant social class and sharing condition.

In the analysis, baseline self-conscious emotion was a significant predictor of self-conscious emotion at time 2 β = 0.71, *t*(93) = 10.18, *p* < 0.01 with lower self-conscious emotion at baseline predicting lower self-conscious emotion at time 2. Social class was not significantly associated with self-conscious emotion change *β* = –0.12, *t*(93) = –1.74, *p* = 0.09, but the pattern of results was in line with the tendency for lower-class individuals to report higher levels of negative self-conscious emotion. The sharing condition was not associated with changes in self-conscious emotion β = –0.05, *t*(93) = –0.76, *ns*. However, these results were all qualified by a significant interaction between social class and sharing condition β = 0.15, *t*(93) = 2.15, *p* < 0.05.

**FIGURE 2:**
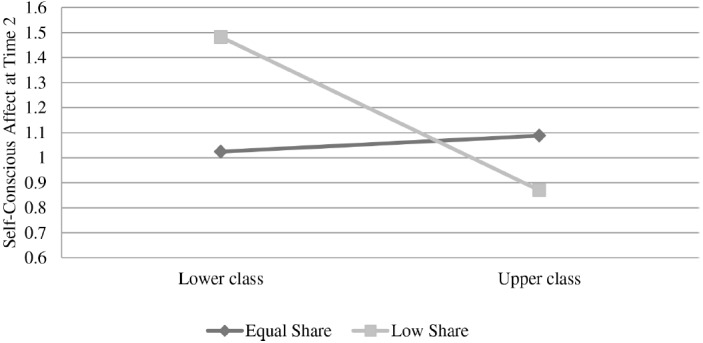
**Negative Self-conscious affect at time 2 as a function of economic sharing condition and participant social class, controlling for baseline negative self-conscious affect (Study 2)**.

The interaction is plotted in Figure 2, and shows a pattern aligning with our prediction: in the equal sharing condition, upper- and lower-class participants show no differences in self-conscious emotion at time 2, *t*(48) = 0.29, *ns*. In contrast, in the low sharing condition lower-class individuals show elevated self-conscious emotion at time 2 relative to their upper-class counterparts *t*(51) = –2.84, *p* < 0.05. Moreover, when adding neuroticism to the analysis as a covariate β = 0.10, *t*(92) = 1.32, *p* = 0.20, the interaction between social class and sharing condition remained significant *β* = 0.15, *t*(92) = 2.21, *p* < 0.05.

We also examined changes in anger and overall negative emotion. In the anger analysis, baseline anger predicted time 2 anger β = 0.56, *t*(96) = 6.64, *p* < 0.01, but no effects were significant *t*s(96) < 1, suggesting that participants’ time 2 anger did not shift based on sharing condition or social class. For overall negative emotion at time 2, only baseline negative emotion was a significant predictor β = 0.74, *t*(94) = 10.97, *p* < 0.01 with participants reporting higher levels at baseline also reporting higher levels at time 2. No other effects were significant *t*s(94) < –1.61. Effects on emotion ratings following the Dictator game appear to be localized to negative self-conscious affect.

### DISCUSSION

The results of Study 2 provide initial experimental evidence aligning with Hypothesis II: exposure to a low, versus equal, share of economic resources within an interaction elicited elevated negative self-conscious affect for lower-class individuals—presumably because such resource interactions remind these individuals of their lower economic standing. In contrast, upper-class individuals showed no such changes in negative self-conscious affect. Interestingly, the findings were specific to negative self-conscious affect and persisted after accounting for trait neuroticism, a correlate of negative affective responses.

## GENERAL DISCUSSION

As economic inequality increases in the United States, people at the bottom of the social class hierarchy face social and economic burdens that include increased physical health problems and reduced access to important social institutions. In addition to these tangible physical and material threats, lower-class individuals also experience a chronic perception that they are lower on the social ladder of society (Kraus et al., 2013b). This subjective sense that one has fewer material and social resources than others is likely to create an additional burden for lower-class individuals—it creates the sense that the self is undervalued relative to that of upper-class individuals.

Two studies provided evidence in support of this general prediction. In Study 1, participants’ lower subjective perceptions of chronic social class rank in society explained associations between lower objective material resource measures of social class and lower self-esteem. In Study 2, exposing individuals to a situation-specific share of low economic resources elicited heightened negative self-conscious affect particularly for lower-class individuals—ostensibly because situation-specific low sharing of resources reminds lower-class individuals of their subordinate status and reduced social and economic value in comparison to others. In both studies, links between perceptions of economic standing and self-evaluation emerged even after controlling for trait measures of neuroticism, which correlate with negative self-evaluation (Judge et al., 2002).

### CAVEATS AND FUTURE DIRECTIONS

The current research aligns with a growing body of theory and evidence suggesting the importance of perceptions of rank in the experience of social class (Adler et al., 2000; Kraus et al., 2012, 2013). Importantly, these findings suggest that it is both chronic and situation-specific perceptions of economic standing—and not necessarily levels of material and social resources—that account for the tendency for lower-class individuals to feel low in self-esteem (Twenge and Campbell, 2002) or to experience guilt in academic contexts (Covarrubias and Fryberg, 2014). As convenience sampling techniques were utilized in these studies, future research with larger representative sampling would do well to further test associations between perceptions of economic standing and self-evaluation.

Interestingly, whereas a low share of resources induced increased self-conscious emotion in lower-class individuals, it did not increase anger. Self-conscious emotions have been shown to compel people to avoid doing things that may lead to personal approbation (Tangney and Dearing, 2003). In addition, of the negative emotions, anger is the one most associated with approach motivational states and action tendencies (e.g., Lerner and Tiedens, 2006). That lower-class individuals saw increases in negative self-conscious emotion—associated with decreases in self-benefitting actions (Tracy et al., 2009)—and no increases in anger following their receipt of a low share of resources leads to the prediction that perceptions of lower-class rank will increase negative self-evaluation and decrease the likelihood that individuals will engage in self-benefitting actions.

Before interpreting the findings of Study 2 more broadly, some limitations in methodology should be highlighted: most critically, our manipulation of resource sharing involved a behavioral check on the effectiveness of the manipulation—low resource sharing elicited less subsequent resource sharing in reciprocation in a second dictator game conducted at the experiment’s conclusion, relative to equal sharing. As this manipulation check is behavioral, it is difficult to interpret the psychological state it induced. For instance, it is unclear if participants experienced a reduced sense of status, felt cheated or unfairly treated, or felt reduced relative economic standing following the manipulation of resource sharing. Though we contend that the manipulation elicited different levels of exposure to resource sharing, the behavioral manipulation check cannot definitely rule out these other compelling alternative explanations.

As well, though we assessed self-evaluative processes using self-conscious affect ratings, a more appropriate design that aligns more directly with Study 1 might involve assessing state self-esteem. Nevertheless, it is interesting to speculate about where the self-conscious emotions reported in Study 2 are derived from: specifically, are relatively lower-class participants who experience a low share of resources actually blaming the self for their partner’s lack of fairness relative to upper-class individuals? Such a finding might suggest fundamental differences between the ways in which people from differing social class standing interpret resource sharing behaviors (see Kraus et al., 2012). Future research is necessary to examine the robustness of this finding before its meaning and implications can be explored.

People of different cultural backgrounds have considerably different ways of thinking about the self, and so it is interesting to speculate about how observed differences in the cultural contexts of relatively upper- and lower-class individuals shape self-evaluation (Snibbe and Markus, 2005; Kraus and Stephens, 2012; Stephens et al., 2012b). Several prior research studies indicate that whereas relatively upper-class individuals tend to behave and perceive the world as separate from the self, relatively lower-class individuals tend to view the self interdependently, as tied to others and the external environment (e.g., Stephens et al., 2007; Kraus et al., 2009). These differences in how the self is culturally defined, in terms of its connection to the external context and other individuals, might change the very meaning of self-esteem and self-conscious emotions themselves. For instance, relatively lower-class rank individuals might report lower levels of self-esteem or more negative self-conscious affect, not because they evaluate the self negatively, but instead, because they diminish the extent that the self stands out above others (for a similar cultural analysis, see Markus and Kitayama, 1991).

This cultural analysis suggests an alternative interpretation to our findings: namely, perceptions of lower-class rank lead individuals to *report* lower self-esteem, not to value the self less than upper-class rank individuals. Aligning with this cultural perspective, future research would benefit from examining whether negative self-evaluations allow lower-class individuals to feel closer to individuals from similar class backgrounds.

Finally, the findings from the current study may help explain some of the current health and social disparities in the United States. For example, the poorer health outcomes experienced by lower-class individuals could, in part, be accounted for by their proneness to experience an increase in self-conscious emotions, as a result of their chronic perceptions of lower rank in the class hierarchy. For example, fifth graders who were shame-prone were more likely to prospectively engage in risky driving behavior and had a lower likelihood of practicing safe sex (Tangney and Dearing, 2003), and there is also a positive link between feelings of shame and substance abuse in adulthood (Dearing et al., 2005; Tangney et al., 2007a). Furthermore, growing research has examined the cultural mismatch that low-income students face when entering higher education (Johnson et al., 2011; Stephens et al., 2012a). As cultural differences are heightened for working class students, who have to encounter the historically upper-class institution on a daily basis, they may experience an increase in self-conscious emotions that leads to academic underachievement through the inhibition of self-benefitting action. Many of these future predictions are worthy of further empirical study.

In this research, we have shown that social class rank shapes how the self is evaluated. The research represents one of the first demonstrations of this phenomenon, and as such, requires further replication and extension before the findings can be applied to new areas of research, or to inform intervention strategies. Notwithstanding these weaknesses, this research suggests that a potentially important way in which social class shapes the self-concept is by enhancing global negative evaluations of lower-class individuals—particularly when those individuals are made aware of their subordinate social class position in a specific situation or context. In this fashion, though lower-class Americans are equal in value to other Americans when considering their capacity to vote or their citizenship, subjective perceptions of social class rank reduce the value of the self for these Americans within their own minds.

### Conflict of Interest Statement

The authors declare that the research was conducted in the absence of any commercial or financial relationships that could be construed as a potential conflict of interest.

## Footnotes

1 We conducted a reverse mediation path analysis to determine if self-esteem explains the association between objective social class and subjective perceptions of social class rank. While the analysis did yield a significant indirect effect *b* = 0.07, SE = 0.03, 95% CI (0.02–0.12) from objective social class to subjective social class rank through self-esteem, the originally significant relationship between objective and subjective social class remained large β = 0.32, *t*(737) = 9.66, *p* < 0.01. These results suggest that our original model using subjective social class rank as the mediator fits the data better than this alternative reverse mediation.
